# Protective effect of transparent film dressing on proton therapy induced skin reactions

**DOI:** 10.1186/1748-717X-8-19

**Published:** 2013-01-24

**Authors:** Jonathan T Whaley, Maura Kirk, Keith Cengel, James McDonough, Justin Bekelman, John P Christodouleas

**Affiliations:** 1 Department of Radiation Oncology, Hospital of the University of Pennsylvania, Perelman Center for Advanced Medicine, TRC 2 West 3400 Civic Center Boulevard, Philadelphia, PA 19104, USA

**Keywords:** Prostate cancer, Proton therapy, Skin toxicity, Radiation dermatitis

## Abstract

**Objective:**

Proton therapy can result in clinically significant radiation dermatitis. In some clinical scenarios, such as lung or breast cancer, the risk of severe radiation dermatitis may limit beam arrangement and prescription doses. Patients undergoing proton therapy for prostate cancer commonly develop mild radiation dermatitis. Herein, we report the outcomes of two prostate cancer patients whose radiation dermatitis appears to have been substantially diminished by transparent film dressings (Beekley stickers).

**Methods:**

This is a descriptive report of the skin toxicity observed in two patients undergoing proton therapy for prostate cancer at a single institution in 2011. A phantom dosimetric study was performed to evaluate the impact of a transparent film dressing on a beam’s spread out Bragg peak (SOBP).

**Results:**

Two patients with low risk prostate cancer were treated with proton therapy to a total dose of 79.2Gy (RBE) in 1.8 Gy (RBE) fractions using two opposed lateral beams daily. Both patients had small circular (2.5 cm diameter) transparent adhesive markers placed on their skin to assist with daily alignment. Patient 1 had markers in place bilaterally for the entirety of treatment. Patient 2 had a marker in place for three weeks on one side and six weeks on the other. Over the course of therapy, both men developed typical Grade 1 radiation dermatitis (asymptomatic erythema) on their hips; however, in both patients, the erythema was substantially decreased beneath the markers. Patient 2 demonstrated less attenuation and thus greater erythema in the skin covered for three weeks compared to the skin covered for six weeks. The difference in skin changes between the covered and uncovered skin persisted for at least 1 month. A phantom study of double scattered beam SOBP with and without the marker in the beam path showed no gross dosimetric effect.

**Conclusions:**

Transparent adhesive markers appear to have attenuated radiation dermatitis in these two patients without affecting the SOBP. One patient may have exhibited a dose–response effect. The reproducibility and underlying mechanisms are unclear. However, the potential to leverage this effect to improve proton-related radiation dermatitis in other clinical scenarios is intriguing. Exploratory animal studies are underway.

## Background

Proton therapy is an attractive radiation modality because plans can be created with relatively low integral dose to normal tissues. However, radiation dermatitis is common because of two features unique to proton plans. First, unlike the large integral dose spread out over a considerable surface area frequently seen with intensity modulated radiation therapy with photons, the physical properties of proton often permit the utilization of fewer beam angles. Second, due to the narrow Bragg peak seen with a monoenergetic beam, proton plans must utilize multiple energies in a given beam direction to create a spread out Bragg peak (SOBP) to cover a target volume with uniform dose [1]. As Bragg peaks of different energies are summed to create uniform dose across the target in depth, there is an increase in entrance dose compared with a monoenergetic Bragg peak due to the summing of the entrance dose from each of the individual peaks. Thus, the limited beam angles and multiple energies utilized to create the SOBP with minimal integral dose can lead to a substantial increase in the entrance dose to skin.

In some clinical scenarios, such as lung or breast cancer, the risk of severe radiation dermatitis may limit proton beam arrangement options and total prescription doses. In patients undergoing proton therapy for prostate cancer, radiation dermatitis is a consistent finding but is usually limited to asymptomatic erythema in the treatment portal. Herein, we report the outcomes of two prostate cancer patients whose radiation dermatitis appears to have been substantially diminished by transparent adhesive markers on the treated skin. In addition, we advance several hypotheses to explain this apparent protective effect.

## Case presentations

This is a descriptive report of the skin toxicity observed in two patients undergoing proton therapy for prostate cancer at the University of Pennsylvania in 2011. Both patients were consented and enrolled onto institutional review board-approved prospective protocols as part of their treatment. Skin toxicity was measured according to the NCI Common Terminology Criteria for Adverse Events v.4 (CTCAE) with asymptomatic, mild erythema representative of grade 1 radiation dermatitis [2].

All patients undergoing proton or photon based radiation therapy for prostate cancer at the University of Pennsylvania have tattoos placed at the time of simulation for daily localization purposes. In a small subset of patients with difficult to identify tattoos, transparent adhesive markers are placed at the time of set-up to aid in daily setup. If these markers detach during a patient’s 9 week therapy, they may be replaced. Beekley transparent markers are approximately 0.1 mm in thickness, 2.5 cm in width, and are made of polyurethane film and acrylate, a common semipermeable film. The film is impermeable to water and bacteria while allowing the diffusion of air to the skin beneath. Neither the thickness nor the composition is uniquely concerning for an interaction with a high energy proton beam.

Both patients in this series were treated for localized low risk prostate cancer with definitive proton therapy. Patient 1 was treated using a double scattering delivery, and patient 2 was treated using uniform scanning delivery. Treatment included 44 fractions of 1.8 Gy (RBE) to a total dose of 79.2Gy (RBE) using two opposed lateral beams daily. After an initial 5040 CGE to the prostate and proximal seminal vesicles, a boost of 2880 CGE was given to the prostate only. Both patients were treated supine in a knee/foot lock for immobilization. Nothing was placed over the skin at the time of treatment. Skin entrance doses ranged from 27 Gy to 29 Gy.

**Figure 1:**
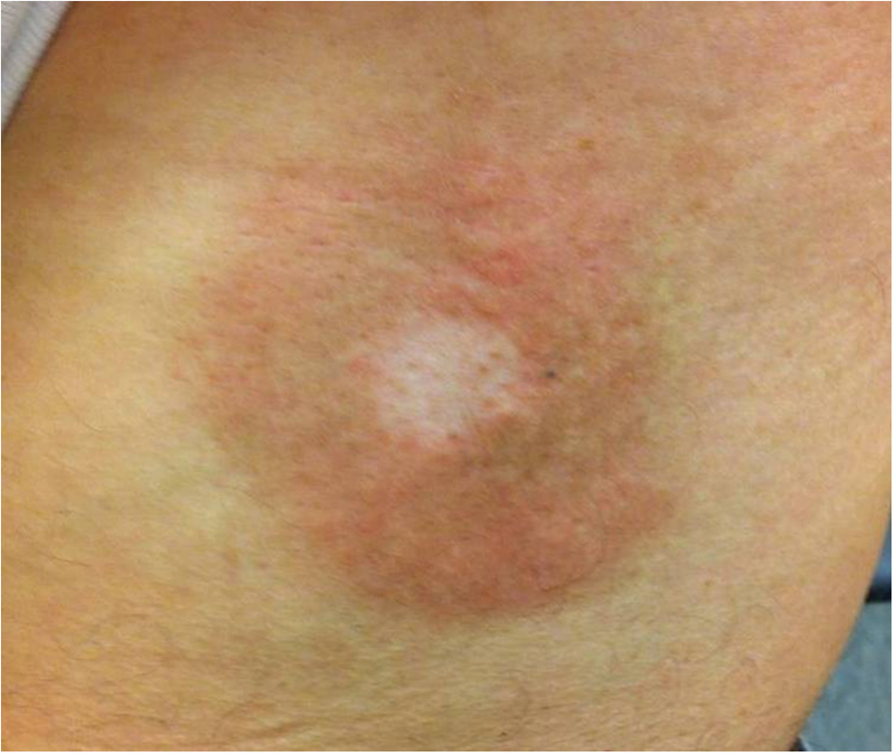
**Diminished radiation dermatitis. **Patient 1 demonstrated substantially decreased erythema in the area of the skin beneath the markers.

Patient 1 had the transparent adhesive marker placed at the time of set-up. The marker was worn throughout treatment and replaced every few weeks to ensure the sticker remained in place. He developed typical asymptomatic erythema on his hips bilaterally; however, the erythema was substantially attenuated in the area of skin beneath the markers (Figure 1). This attenuation was seen and noted to be less pronounced throughout his treatment. His radiation dermatitis was scored as Grade 1. Per patient report, the difference between the erythematous and non-erythematous skin (beneath the marker) persisted for at least 4 weeks. The patient experienced no other acute toxicity during treatment.

**Figure 2:**
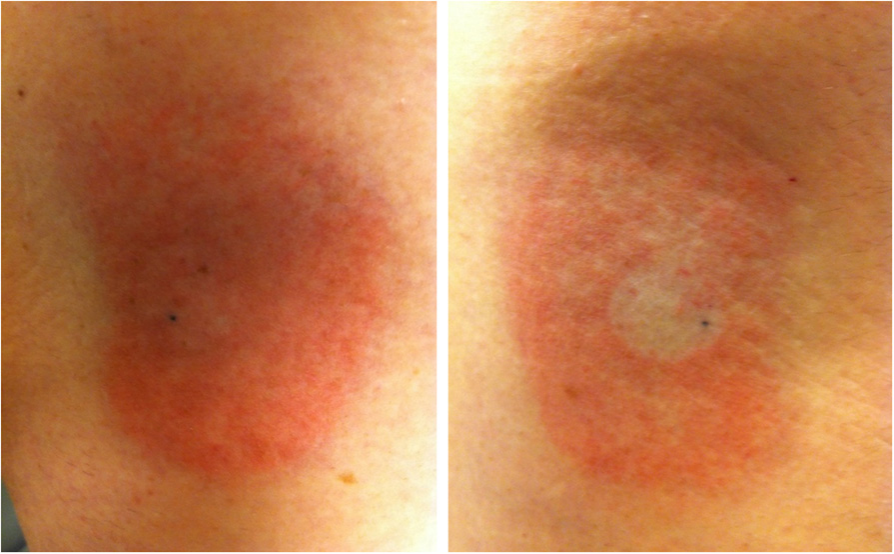
**Dose response effect. **Patient 2 demonstrated more erythema in the skin that was covered for only 3 weeks (Left Image) compared to the skin covered for 6 weeks (Right Image), suggesting a dose response effect.

Patient 2 had the transparent adhesive marker placed at the time of set-up. After 3 weeks, the marker on the left hip detached and was not replaced. The marker on the right hip remained in place for 6 weeks of treatment. He also developed typical asymptomatic erythema on his hips bilaterally. Similar to Patient 1, there was a decrease in skin erythema beneath the adhesive markers. Moreover, the area of skin on the side that was covered for only 3 out of the 6 weeks showed a less noticeable decrease in erythema compared to the surrounding uncovered skin than the area that was covered for 6 weeks (Figure 2). Difference between the erythematous and non-erythematous skin (beneath the marker) persisted approximately 3 months.

**Figure 3:**
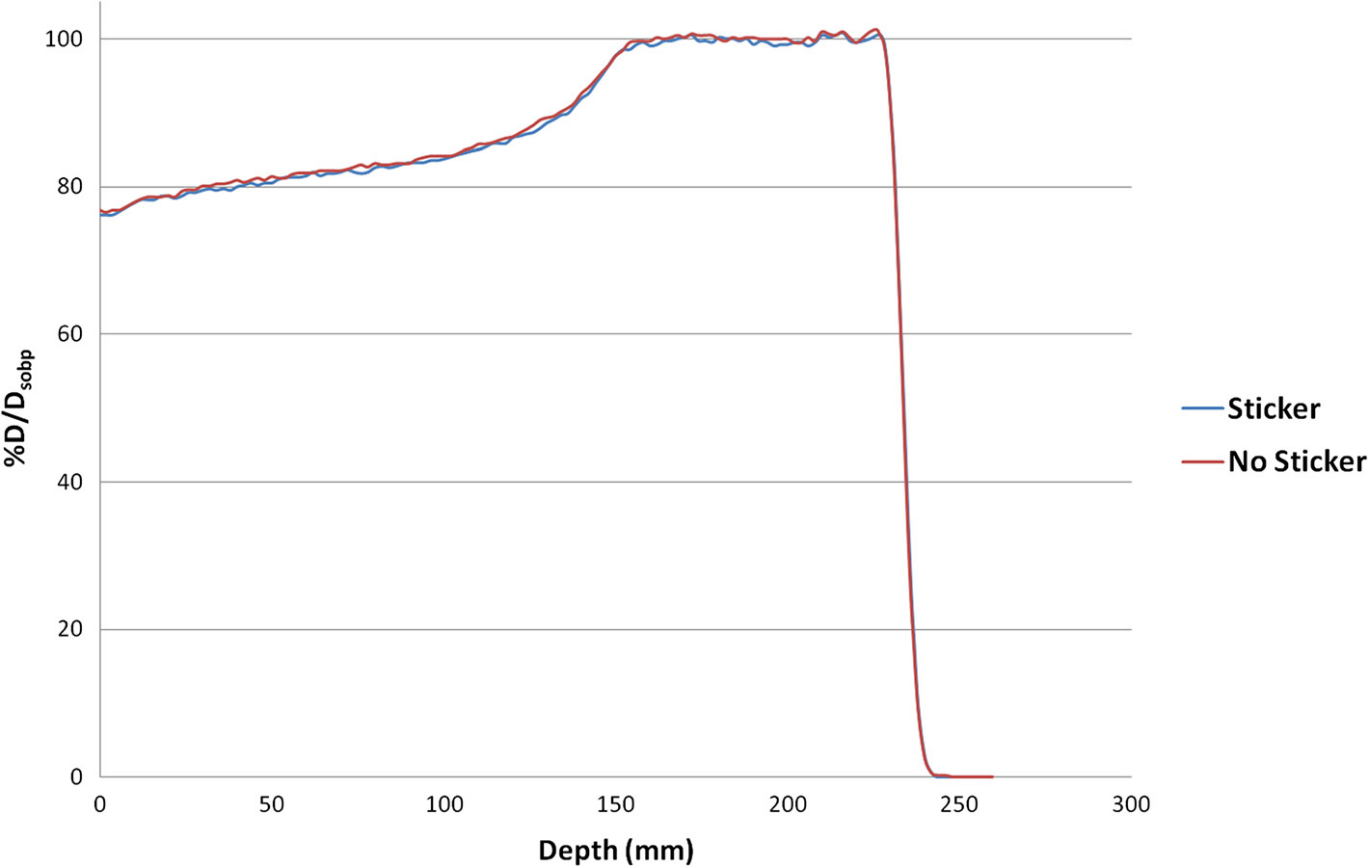
The double scattered SOBP demonstrated no apparent dose variation with and without the transparent marker in the beam.

A gross dosimetric phantom study was performed to assess the effect of transparent adhesive markers, in this case Beekley stickers, on a beam’s SOBP. Using a PPC05 ion chamber in a water tank, two depth dose curves were measured, one with and the other without the transparent adhesive marker placed above the water, centered over the ionization chamber. Double-scattered beams with a 25 cm range and 10 cm modulation width in a 10×10 cm^2^ field were used. These beams are comparable to the treatment fields of the patients considered here which had an average range of 25.4 cm, modulation width of 8.9 cm, and a field radius of 6 cm. The results of the phantom dosimetric study are shown in Figure 3. The plot demonstrates no substantial difference in the SOBPs with or without the marker in place. Data points were sampled every 2mm and have been normalized to the dose in the center of the SOBP. The largest difference in magnitude between data points from the two scans was 0.4% and the nominal range was identical (to a hundredth of a centimeter) for both measurements.

## Discussion

This observational series is the first, to our knowledge, to report an apparent protective effect of transparent adhesive markers on radiation dermatitis associated with proton beam therapy. As an initial investigation step, we performed basic dosimetric calculations on a phantom water tank with adhesive markers that demonstrated no measurable effect on the SOBP in phantom studies. Whether this observation is reproducible and the underlying mechanisms by which it occurred are unclear; however, this phenomenon has been observed in many of our prostate cancer patients undergoing proton therapy. We have used transparent film dressings in patients receiving photon radiation in the past, but due to the lower intrinsic skin dose and larger number of beam angles, patients do not typically have any noticeable skin erythema with treatment. Thus, it is similarly unclear whether this effect is unique to skin effects of proton radiation. Preclinical mechanistic and phenomenological studies are currently underway using a porcine skin model system to test a number of potential hypotheses. These hypotheses separate roughly into two categories, dosimetric and biological.

The possible dosimetric explanations for our observed effect involve the transparent adhesive marker quantitatively altering the physical dose to skin tissue. One such hypothesis is that the transparent adhesive marker disrupts the charged particle equilibrium at the air-skin interface [3]. Interface perturbations are known to occur at air-tissue interfaces and certainly complicate proton treatment planning for lung cancers. However, it is quite unlikely that this particular 0.1 mm marker with a composition comprised mostly of hydrogen and carbon would in any way change the typical air-skin interface. Another hypothesis is that low energy particles are created from scatter from the beam line components upstream of the patient. If the creation of low energy scatter particles were occurring, there is a small possibility these particles could be diminished by 1 mm of substance. Additionally, if the skin reactions were related to creation of low energy particles in the beam line components, we would expect less skin dermatitis in the patients who are treated on the fixed beam gantry that does not have beam line components (i.e. scatterers, MLC, or compensator). However, although we are currently evaluating this using a combination of measurements and modeling, this also seems unlikely given the phenomenon has also been observed with a fixed beam proton gantry.

The possible biological explanations for our observed effect involve transparent adhesive marker altering the skin response to proton radiation. One hypothesis is that the transparent adhesive marker is able to alter the diffusion of oxygen from the external environment, leading to decreased tissue oxygenation in the first few millimeters below the adhesive. The epidermis is known to be devoid of blood vessels and relies on diffusion from capillaries in the papillary dermis for oxygenation [4]. Additionally, it has been suggested that the outermost 0.25-0.4 mm of epidermis is almost entirely oxygenated by external oxygen [5]. If this is in fact the case, small manipulations could alter flow oxygen levels at the level of the epidermis and deep dermis, leading to diminished oxygenation. The mechanism of radiation dermatitis has been documented to involve epidermal atrophy, upper dermal edema, capillary dilation, and melanophage deposition [6] and the slope of the dose response profile for these effects would be decreased if the tissue experienced a modest decrease in oxygen levels. Another hypothesis is that the transparent adhesive marker may alter the tensile forces on the most superficial portions of the skin. As mentioned above, with limited blood supply to the epidermis, small manipulations could alter the supply of inflammatory components from the general circulation. The inflammatory response to radiation is well-documented to involve an influx of inflammatory components [7]. If the tension related to the markers could alter the local response within the most superficial millimeters beneath the markers, it seems reasonable this could relate to the diminished response. Note that these last two mechanisms could act simultaneously to decrease tissue oxygenation. Finally, it is possible that the adhesive could have radioprotective properties. This could occur from direct antioxidant effects that have been observed for topically applied oils and oil derived substances or indirectly through stimulation of mild, acute non-specific local inflammation that has been demonstrated to provide radioprotection through unclear mechanisms [8].

### Potential implications

There are several implications of our observations. First, acute skin toxicity from proton beam has been shown to be dose limiting; therefore, its reduction is an important clinical goal. In a pilot study of proton therapy for accelerated partial breast irradiation in 20 patients with limited beam arrangements, investigators at the Massachusetts General Hospital documented an unexpectedly high rate of acute skin toxicity: moderate to severe skin color changes developed in 79% of patients at 3 to 4 weeks and moderate to severe moist desquamation in 22% of patients at 6 to 8 weeks [9]. In an effort to reduce skin toxicity, investigators at the MD Anderson have proposed an approach using 3–4 beams, though this comes at the expense of exposing a greater volume of non-target tissue [10]. Within our own department, among the patients treated on prospective trials with proton therapy for lung, sarcoma, and previously irradiated recurrent tumors, mild to moderate (Grade II-III) radiation dermatitis is one of the most common toxicities encountered. On occasion, concern for radiation dermatitis has prompted treatment breaks and alteration of beam arrangements.

The potential to leverage the apparent skin-sparing effect described above to improve proton-related radiation dermatitis in other clinical scenarios is intriguing. Exploratory animal studies are underway.

### Consent

According to protocol, written informed consent was obtained from each patient for publication of this report and any accompanying images.

## Competing interests

They authors declare that they have no competing interests.

## Authors’ contribution

JW generated the initial proposal and drafted the manuscript. MK and JM performed dosimetric studies and provided physics theory for the manuscript. KC aided in writing manuscript and providing basis for biological explanations for the observation. JB and JC documented the findings in the patients and provided clinical background for observation. All authors read and approved the final manuscript.

## Funding

National Space Biomedical Research Institute (NSBRI) through NASA NCC 9–58.

## Meeting

This research was presented by Jonathan T. Whaley at 51^st^ Annual Meeting of Particle Therapy Co-Operative Group**,** NCC, Seoul, Korea, May 17–19, 2012.

## References

[B1] Kahn Faiz M The physics of radiation therapy 2010 4 Philadelphia, PA: Lippincott Williams & Wilkins 515 531

[B2] The cancer therapy evaluation program common terminology criteria for adverse events, version 4.0 http://ctep.cancer.gov/forms

[B3] Broerse JJ Zoetelief J Dose inhomogeneities for photons and neutrons near interfaces Radiat Prot Dosimetry 2004 112 4 509 517 10.1093/rpd/nch092 15623886

[B4] Burns DA Breathnach S Cox N Griffiths C Rook’s Textbook of dermatology 2010 8 Malden, Mass: Blackwell Science 3 Blackwell Publishing Ltd. 2010

[B5] Stucker M Struk A Altmeyer P Herde M Baumgartl H Lubbers DR The cutaneous uptake of atmospheric oxygen contributes significantly to the oxygen supply of human dermis and epidermis J Physiol 2002 538 Pt 3 985 994 11826181 10.1113/jphysiol.2001.013067 PMC2290093

[B6] Price NM Radiation dermatitis following electron beam therapy. An evaluation of patients ten years after total skin irradiation for mycosis fungoides Arch Dermatol 1978 114 1 63 66 10.1001/archderm.1978.01640130027008 619784

[B7] Hall Eric J Giaccia Aj Radiobiology for the radiologist 2006 6 Philadelphia, PA: Lippincott Williams & Wilkins 327 348

[B8] Herodin F Laval JD Fatome M Fauve RM Radioprotective effect of an acute non-specific inflammation in mice Int J Radiat Biol Relat Stud Phys Chem Med 1987 51 3 549 559 10.1080/09553008714551021 3494703

[B9] Kozak KR Smith BL Adams J Kornmeh E Katz A Gadd M Spech M Hughes K Gioioso V Lu HM Braaten K Recht A Powell SN DeLan TF Taghian AG Accelerated partial-breast irradiation using proton beams: initial clinical experience Int J Radiat Oncol Biol Phys 2006 66 3 691 698 10.1016/j.ijrobp.2006.06.041 17011445

[B10] Wang X Amos RA Zhang X Taddei PJ Woodward WA Hoffman KE Yu TK Terreffe W Oh J Perkins GH Salehpour M Zhang SX Sun TL Gillin M Buchholz TA Strom EA External-beam accelerated partial breast irradiation using multiple proton beam configurations Int J Radiat Oncol Biol Phys 2011 80 5 1464 1472 10.1016/j.ijrobp.2010.04.052 20708848 PMC3249354

